# Prevalence, risk factors, and infection intensity of fasciolosis in dairy cattle in Boyolali, Indonesia

**DOI:** 10.14202/vetworld.2022.1438-1448

**Published:** 2022-06-12

**Authors:** Heri Kurnianto, Siti Zubaidah Ramanoon, Nor Azlina Abdul Aziz, Soedarmanto Indarjulianto

**Affiliations:** 1 Department of Farm and Exotic Animal Medicine and Surgery, Faculty of Veterinary Medicine, Universiti Putra Malaysia, 43400 Serdang, Selangor, Malaysia; 2 Agriculture Research and Development Agency, Ministry of Agriculture, Indonesia; 3 Department of Veterinary Pathology and Microbiology, Faculty of Veterinary Medicine, Universiti Putra Malaysia, 43400 Serdang, Selangor, Malaysia; 4 Department of Internal Medicine, Faculty of Veterinary Medicine, Universitas Gadjah Mada, Yogyakarta, Indonesia

**Keywords:** dairy cattle, fasciolosis, Flukefinder^®^, Indonesia, prevalence, risk factors

## Abstract

**Background and Aim:**

Fasciolosis is a significant problem in veterinary and public health, causing huge economic losses. Epidemiological studies of fasciolosis in dairy cattle in Indonesia are few and existing reports primarily focus on prevalence. This study aimed to determine the prevalence, risk factors, and infection intensity of fasciolosis in dairy cattle in Boyolali, Indonesia.

**Materials and Methods:**

This cross-sectional study included 400 dairy cattle from 72 household farms in eight subdistricts. Fecal samples (n=400) were examined using the Flukefinder^®^ kit and the simple sedimentation technique was the gold standard for fasciolosis. In-person interviews using questionnaires collected data on farmers, farms, and animal characteristics. Chi-square and logistic regression analyses were performed to evaluate the associated risk factors for fasciolosis, and p < 0.05 was considered statistically significant.

**Results:**

The overall prevalence of fasciolosis in dairy cattle in Boyolali, Indonesia, was 16.50% (95% confidence interval [CI] 12.85-20.15) at the animal level (n = 400), whereas 40.28% at household farms (n = 72) level (95% CI 18.67-51.88). The relative sensitivity and specificity of the Flukefinder^®^ kit compared with those of the gold standard were 79.49% and 92.52%, respectively, with a moderate agreement (kappa=0.59; p < 0.001). Fasciolosis was more likely in cattle originating from the Mojosongo subdistrict than from other subdistricts (odds ratio (OR)=5.28, 95% CI 1.22-22.94); from farms that did not process manure versus from those that did (OR = 3.03, 95% CI 1.43-4.71); and with farmers that had never attended extension programs compared with those who had (OR = 4.72, 95% CI 1.99-11.19). Studied cattle were mostly affected by light *Fasciola* spp. infections (92.4%, 95% CI 77.8-100%) followed by moderate (6.1%, 95% CI 0-22.2%) and heavy (1.5%, 95% CI 0-5.6%) infections.

**Conclusion:**

Fasciolosis is prevalent in dairy cattle in Boyolali, Indonesia. Control efforts should target the high-risk Mojosongo subdistrict, emphasize the importance of processing manure, and encourage farmers to attend extension programs. Flukefinder^®^ is a practical on-site diagnostic kit for fasciolosis in Indonesian dairy farms. Parasite species identification and a malacological survey of intermediate hosts of *Fasciola* spp. in the farming environment are required for further research.

## Introduction

Fasciolosis is a plant-borne parasitic disease caused by trematode parasites *Fasciola hepatica* and *Fasciola gigantica* [1]. These parasites are widespread worldwide and cause huge economic losses in ruminant production [2]. Animals are infected by ingesting freshwater plants contaminated with metacercariae. Cattle are usually infected during grazing or when fed green fodder from agricultural waste such as irrigated paddy rice. Millions of ruminants worldwide have been infected, resulting in an annual economic loss of over US$ 3.2 billion [2]. *F. gigantica* is particularly common in tropical regions [3], including Southeast Asian countries such as Indonesia [4], Cambodia [5], Vietnam [6], Malaysia [7], Lao PDR [8], Thailand [9], Myanmar [10], and the Philippines [11]. The parasites have a complex life cycle involving the snail as an intermediate host [12] and are influenced by environmental variables [13]. Infected animals often show delayed growth, decreased meat and milk production, reduced carcass quality, infertility, anemia, abortion, and death [3, 14–17]. *Fasciola* spp. is a serious public health issue affecting an estimated 2.4-17 million individuals worldwide [18–20]. Conclusive diagnosis of fasciolosis in live animals is generally achieved through microscopic examination to detect fluke eggs in the feces, sedimentation, and the commercial Flukefinder^®^ kit (Richard Dixon ID, United States; http://www.flukefinder.com/) [21, 22]. Despite their limited sensitivity, these techniques have high specificity and are easy to perform, non-invasive, and yield immediate results [23].

Fasciolosis is an endemic disease in cattle and buffalo farms in Indonesia, with a reported prevalence of 4-90% between 2009 and 2020 [24–32]. Economic losses of up to US$ 107 million due to fasciolosis in ruminant production in Indonesia have been reported [33]. Animal husbandry and the agricultural cycle are crucial factors in the epidemiology of fasciolosis in developing countries [34]. Several studies have reported that animal characteristics such as sex, age, and breed; livestock management; area elevation; and climate are significant risk factors for bovine fasciolosis [16, 35–43]. *Boss taurus*, specifically the Friesian breed, is more susceptible to fasciolosis than *Boss indicus* [44], and Friesian Holsteins are 2.63 times more likely to have the disease than Friesians or Holsteins [35].

Boyolali is a dairy farming center in Indonesia and plays a significant role in the domestic dairy industry. The rural area is also associated with crop farming. Most farmers in the area use agricultural byproducts or waste as fodder and manure as crop fertilizer. Epidemiological studies of fasciolosis in Indonesian dairy cattle are few, and there is a risk of underreporting. Available reports primarily focus on disease prevalence.

This study aimed to identify the prevalence, risk factors, and the infection intensity of fasciolosis in dairy cattle in Boyolali, Indonesia. We also evaluated the diagnostic performance of Flukefinder^®^.

## Materials and Methods

### Ethical approval

This research was approved by the Institutional Ethical Committee of the Indonesian Agency for Agricultural Research and Development, Ministry of Agriculture, Indonesia, with reference number Balitbangtan/BPTP Jateng/Rm/01/2020.

### Study period and area

**Figure-1:**
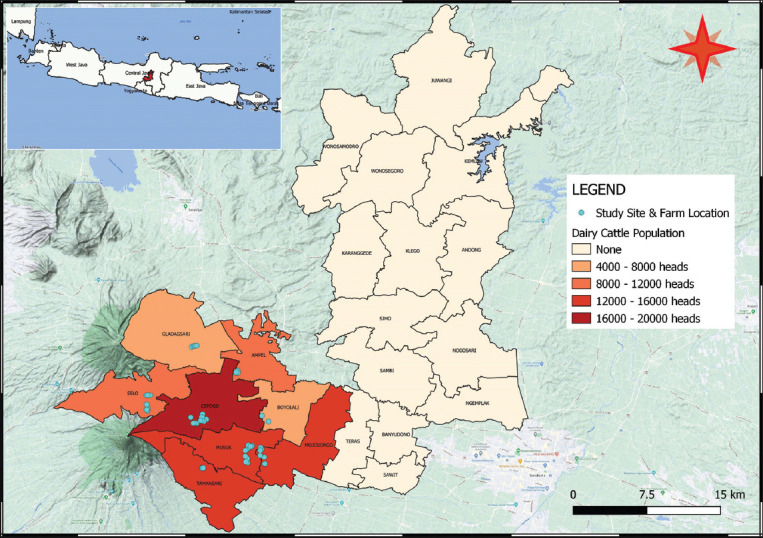
Study area involving eight subdistricts and dairy cattle population in Boyolali Regency of Indonesia [Source: Raw map was sourced from Statistic Bureau Boyolali, and the Figure was created using QGIS version 3.10 A Coruña].

The study was conducted from February to July 2020. The study covered the entire dairy farming area in Boyolali, involving eight subdistricts. Study farm located in ordinate 7°27’25.55” to 7°52’63.7” south latitude and 110°30’19.77” to 110°57’72.7” east longitude with elevation from 506 to 1557 m above sea level (Figure-1). The area lies on the slope of two mountains (Mt. Merapi and Mt. Merbabu) and it is characterized by dry cultivated land, wet climate with an annual rainfall of 1879-2384 mm, air temperature 28.6-32.9°C, and relative humidity 49-92%.

### Study design and sampling

A cross-sectional study was carried out. The total population of dairy cattle in 2019 was 92,601 heads [45]. A sample size of 385 was generated through the Epitools online calculator sample size for a single proportion [46], assuming an expected prevalence of 50%, a confidence level of 95%, and 5% precision. However, the present study involved a total of 400 cattle from 72 household farms located in eight subdistricts (Figure-1): Ampel (n = 9), Cepogo (n = 16), Musuk (n = 12), Gladagsari (n = 6), Tamansari (n = 8), Mojosongo (n = 8), Selo (n = 11), and Boyolali (n = 2) involving eight villages. Villages from each subdistrict were selected randomly. Household farms and animals aged ≥12 months were selected from the villages by convenience. The total number of samples in each village was determined based on probability proportional to the size of the population.

### Coprological examinations

Fecal samples were collected directly from the rectum of each animal, kept in small plastic bags with 10% formalin as a preservative, and stored at 4°C until examination. The eggs of *Fasciola* spp. were identified by the sedimentation technique [47], and Flukefinder^®^ was used to isolate the trematode eggs by differential sieving and sedimentation according to the manufacturer’s instructions. Flukefinder^®^ is a commercial kit that consists of a unit made up of 2″ wide sieves with a predetermined mesh size (the exact size is proprietary to the manufacturer). Identification of egg morphology was made by reference to the literature [48]. Animals with at least one egg in the fecal sample were considered positive for fasciolosis, and at least one positive cow for fasciolosis would indicate a farm was positive. The positive samples were further examined to determine the eggs per gram (epg) by dividing the total number of eggs by the total weight of the sample in grams [49].

### Data collection

In-person interviews using a questionnaire were conducted to collect herd-level data such as farmer sociodemographic, herd size, and husbandry management practice. Farmers’ educational backgrounds were classified into higher (middle school through university) and basic (elementary school or lower), and informal education related to participation in extension programs by government or private. Cattle management practices were assessed related to rice straws feeding, source of drinking water, feed storage facility, deworming schedule, and manure management. A form was used to directly record individual animal data: Age, parity, anemia status, jaundice status, fecal consistency, body condition score (BCS), and submandibular edema. The age of cattle was predicted based on dentition or confirmed using farm records or interviewing the farmer. Cattle were then sorted by age as <2 or ≥2 years. Furthermore, the detail of the parity status was confirmed using farm records and or asking the farmer. The level of anemia was checked through the FAMACHA^©^ (FAffa MAlan CHArt, invented by Dr. Francois “Faffa” Malan of South Africa) eye color chart (1-5) to score the ocular mucous membrane [50] and grouped by anemia (score 3-5) and not anemia (score 1, 2). Jaundice was observed clinically by a veterinarian who identified the condition by yellow discoloration of the skin, sclera, and mucous membrane. BCS was scored 1-5 [51] and classified as <3 or ≥3, and fecal consistency was categorized as normal, soft, or liquid [52]. Localization coordinates and elevation were recorded through the Altimeter (offline true altitude above sea level) app for Android (Pixel Prose SARL, France). The relative humidity and air temperature were recorded through a Digital Thermo Hygrometer (Isolab Laborgeräte GmbH, Germany).

### Statistical analysis

The database was created in Microsoft Excel, and all statistical analyses were performed using IBM Statistical Package for the Social Sciences statistics version 26 software (IBM Corp. Armonk, New York, United States). A significance value was determined with a 95% confidence interval (CI). Distribution of *Fasciola* spp. infections was analyzed throughQGIS spatial software version 3.10 A Coruña (QGIS Development Team 2019, https://www.qgis.org/).

Descriptive analyses were conducted on cattle and household farm data. Age, herd size, and age of respondents are reported as mean and standard deviation (mean ± standard deviation). The prevalence and epg data were based on the combined results of both techniques for coprological examination. The prevalence at the animal level (expressed in percentage, %) was calculated by dividing the number of positive cattle by the number of cattle sampled. Herd prevalence was the number of positive farms divided by the number of study farms.

A Chi-square test was performed to measure the relationship between risk factors and the prevalence of fasciolosis. Factors that were statistically significant at 5% were included as explanatory variables in the multivariable logistic regression.

The sensitivity and specificity were computed by taking the simple sedimentation technique as the gold standard for the diagnosis of fasciolosis with the formula described in Estuningsih *et al*. [32] as follows:

Sensitivity = TP/(TP+FN)×100

Specificity = TN/(TN+FP)×100

Where, TP stands for true positive, TN for true negative, FP for false positive, and FN for false negative.

Cohen’s kappa (k) statistic was used to determine the level of concordance between diagnostic tests, and the k value was interpreted as described by Thrusfield [53].

The intensity of infection was based on the epg in infected animals and classified as low (≤10 epg), moderate (10-25 epg), and high intensity (≥25 epg) [54]. Mann–Whitney U-tests were used to compare mean epg between methods.

## Results

### Cattle and household farm characteristics

All cattle were Friesian Holstein comprising 82% of females and 18% of males (*n* = 400). About 74% of females were lactating and mostly multiparous (61.6%). The average age was 3.88 ± 2.06 years old. Only one animal had submandibular edema, and none had jaundice. The herd size was 8.82 ± 9.82 heads, the cattle did not have access to pasture, and the most common feeding practices were cut and carry. Of the 72 household farmers surveyed, 38 (52.8%) had higher formal education and 48 (58.3%) attended informal education related to dairy farming. The age of the respondents was 52.08 ± 10.94 years old.

### Prevalence of fasciolosis in dairy cattle

**Figure-2:**
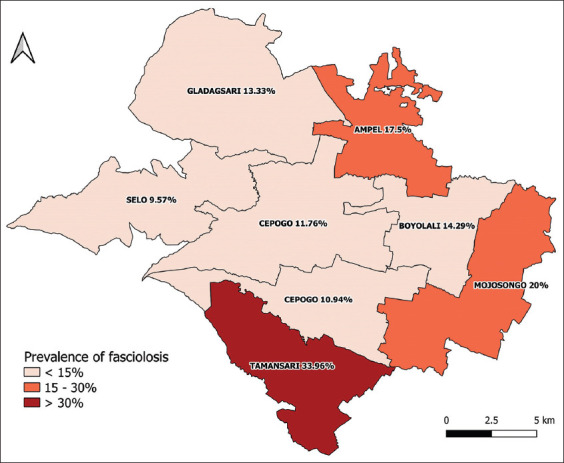
Prevalence of fasciolosis at animal level according to the study area in Boyolali Regency of Indonesia [Source: Raw map was sourced from Statistic Bureau Boyolali, and the Figure was created using QGIS version 3.10 A Coruña].

Of the 400 cattle sampled, 66 cows were positive for fasciolosis based on results from both simple sedimentation and the Flukefinder^®^ kit, resulting in an overall prevalence at the animal level of 16.5% (95% CI 12.85-20.15%). Among 72 household farms, 29 were infected by *Fasciola* spp. The overall prevalence at the herd level was 40.28 (95% CI 28.67-51.88%). According to the simple sedimentation technique alone, the prevalence was 9.75% (95% CI 6.83-12.67%), whereas the Flukefinder^®^ kit indicated 14.50% (95% CI 11.3-17.97%) (Table-1). Figure-2 shows the distribution of fasciolosis prevalence at the animal level according to the study location. The findings of the Chi-square analysis indicated that the prevalence values significantly differed (p < 0.05) by cattle origin, deworming program, processed manure practices, relative humidity, and farmers attending extension programs. Accordingly, a prevalence was noted to be significantly higher in the Tamansari subdistrict compared with that in the other seven subdistricts (Chi-square [χ^2^]=17.07, df = 7, p = 0.017), farms that implemented a deworming program than those that did not (χ^2^ = 7.45, df = 1, p = 0.006), farms that practiced manure processing compared with those that did not (χ^2^ = 12.67, df = 1, p = 0.001), relative humidity of housing area ≥70% than if below 70% (χ^2^ = 5.66, df = 1, p = 0.001), and farms where farmers attended than those that never attended farming-related extension programs (χ^2^ = 10.75, df = 1, p = 0.001). Table-2 summarizes the details of prevalence by variable.

**Table 1:** Prevalence of fasciolosis based on coprological examination of dairy cattle from eight subdistricts of Boyolali Regency, Indonesia.

Coprological examination technique	n	Number of cattle				n	Number of household farms			
	
		Positive	Prevalence (%)	95%CI	p-value*		Positive	Prevalence (%)	95% CI	p-value**
Simple sedimentation	400	39	9.75	6.83-12.67	0.000	72	18	25.00	17.75-35.25	0.000
Flukefinder^®^	400	58	14.50	11.03-17.97		72	26	36.11	24.74-47.48	
Total	400	66	16.50	12.85-20.15		72	29	40.28	28.67-51.88	

p < 0.05 is considered statistically significant.

* χ^2^= 147.210, df = 1;

** χ^2^= 23.197, df = 1

**Table 2:** Results of univariate analysis between potential risk factors and prevalence of fasciolosis in dairy cattle from eight subdistricts of Boyolali Regency, Indonesia.

Factor	Category	n	Number of positive	Prevalence (%)	95% CI	χ^2^	p-value
Total number of samples		400	66	16.50	12.85–20.15		
Origin of cattle	Ampel	40	7	17.50	5.19–29.81	17.073	0.017*
	Cepogo	85	10	11.76	4.77–18.76		
	Musuk	64	7	10.94	3.08–18.80		
	Gladagsari	30	4	13.33	0.42–26.24		
	Tamansari	53	18	33.96	20.78–47.14		
	Mojosongo	65	13	20.00	10.01–29.99		
	Selo	35	3	8.57	1.19–18.33		
	Boyolali	28	4	14.29	0.47–28.10		
Gender	Male	72	13	18.06	8.95–27.16	0.154	0.695
	Female	328	53	16.16	12.15–20.16		
Lactation status	Yes	244	36	14.75	10.27–19.24	1.387	0.239
	No	84	17	20.24	11.47–29.01		
Age	<2 years	65	10	15.38	6.37–24.39	0.070	0.791
	≥2 years	335	56	16.72	12.70–20.73		
Parity	Heifers	53	11	20.75	9.47–32.04	1.621	0.445
	Primiparous	73	9	12.33	4.60–20.05		
	Multiparous	202	33	16.34	11.19–21.48		
Body condition score	<3	209	35	16.75	11.64–21.85	0.019	0.89
	≥3	191	31	16.23	10.95–21.51		
Indication of anemia	Not-anemia	62	15	24.19	13.23–35.16	3.152	0.076
	Anemia	338	51	15.09	11.25–18.92		
Fecal consistency	Normal	87	18	20.69	12.01–29.37	1.896	0.387
	Soft	294	44	14.97	10.86–19.07		
	Liquid	19	4	21.05	0.86–41.24		
Source of cattle	Animal market	323	59	18.27	14.03–18.22	3.799	0.051
	Non-animal market	77	7	9.09	2.52–115.66		
Herd size	≤5 heads	102	23	22.55	14.30–30.80	3.636	0.057
	>5 heads	298	43	14.43	10.42–18.44		
Rice straw given	Yes	233	44	18.88	14.47–24.99	3.818	0.051
	No	177	22	12.43	7.52–17.34		
Feed storage	No	231	45	19.48	14.34–24.63	3.525	0.060
	Yes	169	21	12.43	7.40–17.45		
Drinking water	Regional water/well	289	43	14.88	10.74–19.01	1.986	0.159
	Buy/rain	111	23	20.72	13.06–28.38		
Deworming program	No	243	50	20.58	15.46–25.70	7.446	0.006*
	Yes	157	16	10.19	5.41–14.98		
Manure processed	No	243	53	21.81	16.58–27.04	12.674	0.001*
	Yes	157	13	8.28	3.92–12.64		
Altitude	≤1200 masl	365	63	17.26	13.37–21.16	1.750	0.186
	>1200 masl	35	3	8.57	–1.19–18.33		
Temperature	<30°C	259	42	16.22	11.70–20.74	0.043	0.836
	≥30°C	141	24	17.02	10.74–23.30		
Relative humidity	<70%	168	19	11.31	6.47–16.15	5.664	0.017*
	≥70%	232	47	20.26	15.05–25.47		
Season	Wet	315	56	17.78	13.53–22.02	1.757	0.185
	Dry	75	10	13.33	4.77–18.76		
Age of farmer	≤40 years	80	15	18.75	10.01–27.49	1.100	0.557
	40≤50 years	98	13	13.27	6.43–20.10		
	>50 years	222	38	17.12	12.12–22.11		
Education level	Basic	151	27	17.88	11.70–24.06	0.336	0.562
	Higher	249	39	15.66	11.12–20.21		
Participation in extension program	Never	187	43	22.99	16.91–29.08	10.751	0.001*
	Ever	213	23	10.80	6.60–15.00		
Farmer experience	≤25 years	220	41	18.64	13.45–23.82	1.62	0.203
	>25 years	180	25	13.89	8.79–18.99		

* Statistically significant (p < 0.05), χ^2^=Chi-square, masl=Meter above sea level

### Sensitivity and specificity of Flukefinder^®^ kit and agreement between Flukefinder^®^ kit and simple sedimentation technique

Compared to the simple sedimentation technique, the sensitivity and specificity of the Flukefinder^®^ kit were 79.49% (95% CI 66.66-92.31%) and 92.52% (95% CI 89.81-95.23%), respectively. There was a significant difference between the two methods (χ^2^ = 147.21, df = 1, p = 0.000), k value 0.59 (p = 0.000) (Table-3).

**Table 3:** Comparison between Flukefinder^®^ kit and simple sedimentation technique in the diagnosis of fasciolosis and kappa index value for agreement between tests.

Flukefinder^®^ kit	Sedimentation		Total

	Positive	Negative	
Positive	31 (TP)	27 (FP)	58
Negative	8 (FN)	334 (TN)	342
Total	39	361	400

Chi-square (χ^2^)=147.21, df=1, p *=* 0.000, kappa index=0.59

### Risk factors of fasciolosis in dairy cattle

This study identified three significant risk factors: Cattle origin, processed manure practices, and farmers attending extension programs (Table-4). Fasciolosis was more likely in Mojosongo subdistrict than in other subdistricts (odds ratio [OR]=5.28, 95% CI 1.22-22.94); farms that did not practice manure processing than those farms that did (OR=3.03, 95% CI 1.43-4.71); and farms where the farmers had never attended any extension program than those that had (OR=4.72, 95% CI 1.99-11.19).

**Table 4:** The multivariate logistic regression analysis results for the potential risk factors of fasciolosis in dairy cattle from eight subdistricts of Boyolali Regency, Indonesia.

Factor	Category	B	S.E.	Wald	Sig.	OR	95% CI
Origin of cattle	Gladagsari	0.731	0.927	0.623	0.43	2.077	0.338–12.770
	Boyolali	−0.358	0.658	0.297	0.586	0.699	0.193–2.537
	Ampel	0.045	0.847	0.003	0.957	1.046	0.199–5.499
	Tamansari	0.08	0.759	0.011	0.916	1.084	0.245–4.797
	Selo	−0.318	0.894	0.127	0.722	0.728	0.126–4.195
	Musuk	−0.912	0.775	1.384	0.239	0.402	0.088–1.836
	Mojosongo	1.664	0.75	4.928	0.026*	5.28	1.215–22.944
	Cepogo					Ref	
Deworming program	No	0.682	0.442	2.375	0.123	1.977	0.831–4.705
	Yes					Ref	
Manure processed	No	1.107	0.381	8.424	0.004*	3.026	1.433–6.391
	Yes					Ref	
Relative humidity	≥ 70%	0.995	0.6	2.75	0.097	2.704	0.835–8.764
	< 70%					Ref	
Participation in extension program	No	1.551	0.441	12.385	0.000*	4.717	1.988–11.189
	Yes					Ref	

B=Estimated value, S.E.=Standard error, OR=Odds ratio, CI=Confidence interval, Ref=Reference category.

* Statistically significant (p < 0.05)

### The intensity of *Fasciola spp*. infection

Three categories of fasciolosis infection intensity were based on epg from both coprological techniques. Sixty-one (92.42%) of the 66 cattle sampled were lightly infected, with moderate infection in 6.06% (4/66) and 1.52% (1/66) with heavy infection (Table-5). The mean ( ± Standard error) of epg was 3.95 ± 0.70 (range 1-34). There was no significant difference (p>0.05) in mean epg between the simple sedimentation and Flukefinder^®^ kit methods.

**Table 5:** Intensity of infection with *Fasciola* spp. of dairy cattle from eight subdistricts of Boyolali Regency, Indonesia, according to animal factors and clinical signs (n = 66).

Factor	Category	Frequency	Intensity of infection, frequency (%)		

			Light	Moderate	Heavy
Gender	Male	13	12 (92.31)	1 (7.69)	0 (0.00)
	Female	53	49 (92.45)	3 (5.66)	1 (1.89)
Age	<2 years	10	10 (100)	0 (0.00)	0 (0.00)
	≥2 years	56	51 (91.07)	4 (7.14)	1 (1.79)
Parity*	Heifers	11	10 (90.91)	1 (9.09)	0 (0.00)
	Primiparous	9	7 (77.78)	2 (22.22)	0 (0.00)
	Multiparous	33	32 (96.97)	0 (0.00)	1 (3.03)
Body condition score (BCS)	<3	35	34 (97.14)	1 (2.86)	0 (0.00)
	≥3	31	27 (87.10)	3 (9.68)	1 (3.23)
Indication of anemia	Not anemia	15	14 (93.33)	1 (6.67)	0 (0.00)
	Anemia	51	47 (92.16)	3 (5.88)	1 (1.96)
Fecal consistency	Normal	18	15 (83.33)	2 (11.11)	1 (5.56)
	Soft	44	42 (95.45)	2 (4.55)	0 (0.00)
	Liquid	4	4 (100)	0 (0.00)	0 (0.00)

* Infected female cattle=53 cows

## Discussion

The present study revealed that fasciolosis is prevalent in Boyolali, Indonesia, indicating its importance in dairy farms and rural livelihood. About 95% of the dairy cattle population in Indonesia is in rural communities, and animals are managed by small farms, kept conventionally with a lack of good farming practices [55]. Furthermore, fasciolosis is a common disease in cattle in Indonesia [56] and remains a constraint in ruminant production. Furthermore, the humid tropical weather of Indonesia is a suitable ecology for the life cycle of the disease [57].

The average prevalence of fasciolosis in dairy cattle from our study was higher than that observed in Lampung, Indonesia (12%) [58] but lower than in Malang, Indonesia (58%) [59]. The discrepancies in prevalence across the three locations might be explained by environmental conditions, farm management systems, and diagnostic methods. In addition, our research recorded a lower prevalence of liver fluke infection in Indonesian beef cattle [24, 28,59]. Nevertheless, it was comparable with the Indonesian buffalo [25]. Compared with other countries in Southeast Asia, our research recorded a prevalence higher than in Thailand (4%) [60] and lower than in Vietnam (34%) [61]. In general, dairy cattle in Indonesia are kept more intensively than beef cattle farming, explaining the different prevalence levels. In intensive farming systems, animals are permanently housed, limiting their exposure to grass contaminated with parasites compared with grazed animals. Keyyu *et al*. [62, 63] reported that fasciolosis was associated with the type of management system, and traditional systems create a significant risk for disease prevalence. Likewise, variations in prevalence among countries could be explained by differences in farmer knowledge, farming practices, and environmental and climate conditions [2]. Indeed, parasites require a suitable environment, humidity, and temperature [64].

Moreover, host, snail distribution, and diagnostic procedures may also influence the occurrence of fasciolosis [12, 65–67]. Host, sex, age, breed, and BCS have all been considered predictors of fasciolosis prevalence [35, 66]. The distribution of snails was consistent with liver fluke infection in cattle; for example, *Lymnaea natalensis* in Iringa District, Tanzania, was directly associated with the prevalence of fasciolosis caused by *F. gigantica* and elevation of studied areas [68]. However, the abundance of cercariae of *F. gigantica* in snail populations is not a reliable predictor of prevalence in the definitive hosts [57]. Unfortunately, this study did not provide information on the distribution of intermediate host and the presence of liver fluke cercariae; therefore, a malacological survey should be undertaken. There are various diagnostic tests for fasciolosis, including microscopic egg identification, antibody and antigen testing, and molecular detection, all of which have varying sensitivity and specificity [22]. This study identified the eggs of *Fasciola* spp. but could not confirm the worm species. Fasciolosis in Indonesia has been caused by *F. gigantica*; however, a survey by Prasetya *et al*. [69] found a hybrid form in Kalimantan Island, Indonesia. It was suspected to be similar to *Fasciola* from Iran. Thus, neither morphometry nor molecular methods to identify species are very meaningful because introgressed forms may exhibit enhanced pathogenicity and virulence [70]. These introgressions are also related to the broadly reported anthelminthic drug resistance of *F. hepatica* and *F. gigantica* [71].

The present study compared the diagnostic performance of the Flukefinder^®^ kit with a simple sedimentation technique for the diagnosis of fasciolosis in dairy cattle. This test kit principally adopts the sedimentation process, and this tool is considered new in Indonesia. Our findings were comparable with a previous study conducted by Howell [72] in which this kit was compared with gross liver inspection, which has a sensitivity of 64.3% (95% CI 35.1-87.2) and specificity of 85.0% (95% CI 62.1-97.8).

The moderate test agreement between the Flukefinder^®^ kit and the simple sedimentation technique [53] was also consistent with a previous study conducted by Reigate *et al*. [23]. The Flukefinder^®^ kit was suitable for accurate diagnosis based on its sensitivity and specificity, and ability to recover 100% of eggs at a low threshold of ≥5 epg [23]. According to Rojas *et al*. [22], many laboratories routinely apply the conventional technique of fecal sedimentation to detect fasciolosis. Those techniques are affordable, simple to use, and readily applicable in the field for rapid detection, despite their limited sensitivity and suitability for usage throughout the chronic phase [22]. Aside from low sensitivity, Flukefinder® technically takes longer in the sedimentation process if the sample size exceeds the manufacturer’s recommendations; actually, using more feces would increase the sensitivity [21].

The Mojosongo subdistrict was a significant risk factor for fasciolosis in dairy cattle. Mojosongo lies at a lower elevation than other study locations, and irrigated paddy fields are nearby. Farmers in the area might have utilized agricultural waste, especially rice straw, as fodder for their cattle more frequently. Suhardono *et al*. [73] revealed that fresh rice stems from irrigated fields are a source of liver fluke infection in cattle. Rice straw is contaminated with the metacercaria of *Fasciola* spp. carried by the intermediate hosts. Two freshwater snails (*Lymnaea rubiginosa* and *Indoplanorbis* spp.) in the surrounding farming and swamp area have been identified as inter­mediate hosts for *F. gigantica* in Indonesia [57, 74]. The agriculture area shares similarities with Mojosongo and may explain why it was a high-risk area for fasciolosis in Boyolali.

This study found that cattle from farms that did not handle manure were more likely to have fasciolosis. In rural agriculture, farmers frequently use raw manure as crop fertilizer and collect agricultural waste for fodder. This farming cycle is an essential factor in the parasite’s life cycle. Conversely, proper management of cattle feces can limit parasite transmission; for instance, composting or drying could inhibit egg development since increased temperature and heating time causes egg fatality [75] as they cannot survive temperatures over 43°C [57]. The eggs would die quickly because composting cattle manure occurs at a nearly constant 50°C for approximately 30 days [76], depending on the material composition and composting procedure applied. In addition, infective larvae perish when exposed to sunlight for 8 h [77]. Therefore, drying manure and fodder before use effectively terminate the parasite’s life cycle. This study revealed that liver fluke infection in the farms that processed manure was significantly lower than in farms that did not practice manure processing.

Farmers who have attended animal husbandry extension courses were less likely to have liver fluke infections among their herds. This result could be explained by the fact that farmers exposed to the programs would be more conscientious about their animal health and disease prevention. The Indonesian government regularly conducts extension programs to enhance farmers’ knowledge of livestock management, feed, reproduction, and animal health. Information regarding fasciolosis is an important part of the program since the disease is commonly reported in animals sacrificed during Eid al-Adha [30, 78]. Extension programs may become a useful instrument for disseminating information to rural communities, and it is a powerful medium for increasing farm productivity [79, 80]. Moreover, it has a considerable influence on improving living conditions, especially for rural dairy farmers [81]. It might be inferred that extension activities could encourage farmers to be more aware of their cattle and farming practices, particularly efforts to avoid economic losses due to diseases.

The intensity of infection in our study was determined based on the total number of epg in feces, and the data revealed a low infection rate in the animals studied. The mean epg was higher than a previous study conducted in Malang, Indonesia (1.88) [82], perhaps due to a low fluke population in the liver of infected cows. There was not always a linear relationship between infected cattle and the presence of *Fasciola* spp. eggs in the feces, as reported by Anderson *et al*. [83], who only discovered eggs in the feces of 70% of afflicted animals. Molina *et al*. [84] found a correlation between the number of adult flukes and egg counts. The capacity of *Fasciola* spp. to survive and infect a host depends on the interaction between host and parasite factors, which are related to immunological systems [85]. Lalor *et al*. [70] revealed that the ability of the parasite to establish infection in a mammalian host depends on its ability to manipulate the host’s physiological milieu by producing and releasing a complex of regulatory proteins, glycans, and microRNAs. The parasite’s tegument also serves a vital function in defending it from assaults by the host immune system [70]. Subsequently, the low epg value might be attributed to the sedimentation technique used and the weight of the feces tested [64]. Reigate *et al*. [23] reported that the Flukefinder^®^ kit was consistently sensitive (100%) when there were at least five epg in a fecal sample versus a minimum of 10 epg required for simple sedimentation, with egg recovery rates of 38% and 5%, respectively.

The study findings indicated that liver fluke infection is present in all dairy farm locations in Boyolali, Indonesia. We believed that the parasite was transmitted by green fodder derived from agricultural waste collected by farmers from lowland fields. The adjacent dairy farming area is defined as a dry agricultural environment where there is likely little to support the life cycle of *Fasciola* spp. However, further surveys of the snail population are necessary. Furthermore, traditional farming management usually demonstrates a lack of disease awareness whereby the rural animal housing is mainly located around or linked to the farmer’s house; consequently, the farmers might be contaminated or infected by the parasite when handling their animals. Indeed, Indonesian *Fasciola* has been shown to infect humans [86–88]. Fasciolosis has a high risk of becoming a serious public health concern in Boyolali dairy farming communities if no attempt is made to improve farmers’ knowledge of mitigating the risk of infection.

## Conclusion

This study revealed that fasciolosis is prevalent in dairy cattle farming in the Boyolali Regency of Indonesia. The prevalence of fasciolosis varied among subdistricts, despite mostly showing light infection. Significant risk factors identified in the present study were animal origin, the implementation of manure processing in farms, and farmers attending extension programs. Furthermore, the Flukefinder^®^ kit is a valuable diagnostic tool for fasciolosis and is practical for on-site use. This study has not covered liver fluke species and the distribution of the intermediate host of *Fasciola* spp. and its relationship with the prevalence; hence, species identification and a malacological survey will be necessary for future investigation. Our study findings provide baseline epidemiological information and could be used to develop and implement a control and prevention program for fasciolosis in dairy farming in Indonesia.

## Authors’ Contributions

HK: Designed the study, investigation, data analysis, visualization, writing – original draft, and editing the manuscript. SZR: Supervision, designed the study, data analysis, and review and editing the manuscript. NAAA and SI: Supervision, designed the study, and review of the manuscript. All authors have read and approved the final manuscripts.
